# 

**Published:** 1892-01

**Authors:** 


VOL. XIII.] *PUBLISHED MONTHLY AT THREE DOLLARS A YEAR. SINGLE COPIES, THIRTY CENTS.* [NO. I.

Entered at the Post Office, February 3rd, 1891, at New York. as Second-class Matter.
Copyright, 1891, by W. A Conklin and R. S. Huidekoper.
Printed for the Editors by E. SCOTT, 134 West 23d Street, New York.

### THE JOURNAL OF COMPARATIVE MEDICINE AND VETERINARY ARCHIVES.

EDITED BY

W. A. CONKLIN, Ph. D., D. V. S.,
*Director of Zoological Gardens, New York.*

RUSH SHIPPEN HUIDEKOPER, M. D., Veterinarian, (*Alfort.*)

*Professor of Sanitary Medicine and Veterinary Jurisprudence,*

*American Veterinary College,*

*New York.*

SURGEON GENERAL'S OFFICE, LIBRARY.

JANUARY, 1892.

All communications and payments should be addressed to

**MANAGING EDITOR JOURNAL COMP. MEDICINE AND VET. ARCH.**

FORTY-SECOND STREET AND PARK AVENUE,

NEW YORK.

LONDON: BALLIERE, TINDALL & COX.

